# A systematic literature review of disability weights measurement studies: evolution of methodological choices

**DOI:** 10.1186/s13690-022-00860-z

**Published:** 2022-03-24

**Authors:** Periklis Charalampous, Suzanne Polinder, Jördis Wothge, Elena von der Lippe, Juanita A. Haagsma

**Affiliations:** 1 Department of Public Health, Erasmus MC University Medical Center, Rotterdam, The Netherlands; 2 German Environment Agency, Section Noise Abatement of Industrial Plants and Products, Noise Impact, Wörlitzer Pl. 1, 06844 Dessau-Roßlau, Germany; 3 Department of Epidemiology and Health Monitorin, Robert Koch Institute, Berlin, Germany

**Keywords:** Burden of disease, Disability weight, Disability adjusted life years, Valuation of health states

## Abstract

**Background:**

The disability weight is an essential factor to estimate the healthy time that is lost due to living with a certain state of illness. A 2014 review showed a considerable variation in methods used to derive disability weights. Since then, several sets of disability weights have been developed. This systematic review aimed to provide an updated and comparative overview of the methodological design choices and surveying techniques that have been used in disability weights measurement studies and how they evolved over time.

**Methods:**

A literature search was conducted in multiple international databases (early-1990 to mid-2021). Records were screened according to pre-defined eligibility criteria. The quality of the included disability weights measurement studies was assessed using the Checklist for Reporting Valuation Studies (CREATE) instrument. Studies were collated by characteristics and methodological design approaches. Data extraction was performed by one reviewer and discussed with a second.

**Results:**

Forty-six unique disability weights measurement studies met our eligibility criteria. More than half (*n* = 27; 59%) of the identified studies assessed disability weights for multiple ill-health outcomes. Thirty studies (65%) described the health states using disease-specific descriptions or a combination of a disease-specific descriptions and generic-preference instruments. The percentage of studies obtaining health preferences from a population-based panel increased from 14% (2004–2011) to 32% (2012–2021). None of the disability weight studies published in the past 10 years used the annual profile approach. Most studies performed panel-meetings to obtain disability weights data.

**Conclusions:**

Our review reveals that a methodological uniformity between national and GBD disability weights studies increased, especially from 2010 onwards. Over years, more studies used disease-specific health state descriptions in line with those of the GBD study, panel from general populations, and data from web-based surveys and/or household surveys. There is, however, a wide variation in valuation techniques that were used to derive disability weights at national-level and that persisted over time.

**Supplementary Information:**

The online version contains supplementary material available at 10.1186/s13690-022-00860-z.

## Background

The Disability-Adjusted Life Year (DALY) is a population health metric that measures the burden of disease of a population by integrating mortality in Years of Life Lost (YLL) and morbidity in Years Lived with Disability (YLD) [1–3]. It was first used in the early 1990s, in the first iteration of the Global Burden of Disease and Injury (GBD) study; a landmark global effort to estimate fatal and non-fatal health outcomes using a health metric that allows comparisons of the impact of different diseases, injuries, and risk factors over time and between geographies [4–6]. Thus, the DALY-concept provides a comprehensive health overview and is a crucial tool in facilitating decision-making on disease prevention.

The disability weight is an essential factor to assess DALYs, and in particular to estimate the healthy time that is lost due to living with a certain state of illness [7]. A disability weight is a weighting factor that reflects the relative severity of a health state, with a value anchored from 0 to 1, with 0 implying a state that is equivalent to full health and 1, a state equivalent to death. The first set of disability weights was established for the GBD 1996 study [8]. Since then, multiple alternative sets of disability weights have been developed, each using different design choices [9]. A set of disability weights refers to a collection of disability weights that resulted from one specific disability weight study.

The disability weight is a so-called social value; it is based on preferences of a certain population [7, 10]. This population can consist of, for instance, persons of the general population or a group of health professionals [7]. The characteristics of the persons who provide the preferences have implications for the description of the health state and for the difficulty of the health state valuation tasks that are used to elicit the preferences for health states. These health state valuation tasks can consist of a relatively simple task of choosing the healthier person out of two, or much more complicated tasks that require the respondent to make a trade-off between two hypothetical scenarios of health programs that emulate health policy decisions [7, 11]. Notably, the GBD 1996 set of disability weights [8] was based on the health state valuations of a group of 10 health professionals that evaluated disease labels for 483 sequelae resulting from 131 diseases and injuries (e.g., “dislocation of shoulder: long term, with or without treatment”) without a further description of symptoms or physical impairments, whereas the GBD 2010 set of disability weights [12] was based on the health state valuations of more than 30,000 persons from the general population evaluating short disease descriptions for 220 unique health states without a disease label (e.g., “has a shoulder that is out of joint, causing pain and difficulty moving. The person has difficulty with daily activities such as dressing and cooking”).

In 2014, an overview of disability weight studies and their design choices was published [9]. However, since then several other disability weights measurement studies have been performed, either because a national burden of disease study was performed, with the researchers preferring to use disability weights that are based on the preferences of the national population [13–16] or because disability weights for certain diseases were unavailable [17–19]. Another reason may be that existing disability weights were too granular, meaning that the disability weights represent health states that are heterogeneous with respect to the severity level of functional limitations [12, 20], and may therefore hamper the mapping of disability weights to available epidemiological data.

Therefore, this systematic literature review aimed to provide an updated and comparative overview of the methodological design choices that have been used in disability weights measurement studies. The following research questions were addressed:

- How many disability weights measurement studies have been conducted, and in which countries?
- Which methodological design choices have been used to describe and value health states in disability weights measurement studies and how did these evolve over time?

## Methods

### Methodological design choices in disability weight studies

There are five methodological aspects of estimating disability weights for different states of health. The first design choice relies on the health state description. The health state can be described using a generic or a disease-specific method. A generic health state description indicates the functional health status regardless of the underlying health condition [21, 22]. Multi-attribute utility instruments can be used to generate generic health state descriptions. With multi-attribute utility instruments, generic attributes are used to classify health states; for each health state a functional level is chosen for each attribute. To classify health states, several generic instruments are available, such as the EQ-5D [23] or SF-36 [24] health questionnaires, or a combination of these attributes namely Classification and Measurement System of Functional Health (CLAMES model) [25]. Using weights for the separate attributes, the reported functional level on the attributes is then converted into a disability weight which by definition fits within the 0–1 range. A disease-specific health state description indicates the cause and/or the functional consequences and symptoms associated with the condition [21]. A health state description that combines generic and disease-specific health state is also used [26].

The second design choice involves the panel of judges. In essence, the values of disability weights are usually assigned based on the preferences of medical experts [11], health professionals [11], patients or people with disabilities [11], representative population samples [11], or a combination of these groups [11, 27].

The third design choice relates to the valuation methods for health states. Several measurements exist, of which the visual analogue scale (VAS), interpolation, time trade-off (TTO), person trade-off (PTO), standard gamble (SG), paired comparison (PC), and population health equivalence (PHE) have been widely applied to measure individual preferences [11, 22]. The VAS valuation method requires participants to score a health state of disease on a vertical, calibrated line graded from 0 (“worst imaginable health state”) to 100 (“best imaginable health state”). The interpolation technique requires the panel members to value health states by placing each health state of disease as similar to or in-between indicator health states on the calibrated disability scale [26, 28]. The TTO method elicits preferences for states of health by asking participants to choose between a certain amount of time in the presented health state or a shorter life spent in full health. The PTO method asks respondents to trade-off numbers of person-years living in good health and person-years lived in a lesser state of health. The SG method asks respondents to make choices that weigh health improvements against risk of death. With the PC technique, two alternative health states are presented and the respondents have to decide which is more desirable. The PHE technique requires participants to compare health benefits of different health programmes. Each of these tools has advantages and disadvantages. Information about the advantages and disadvantages of these valuation techniques have been described elsewhere [11, 29].

The fourth design choice relates to the time presentation. Disability weights of the health states can be subdivided into annual health profile and/or period profile disability weights. The annual profile approach describes the course of the health state over a 1-year period, whereas the period profile approach assumes that the duration of the health state remains constant over time [7, 30]. However, the annual profile approach has been previously suggested to assess disability weights for conditions with acute onset or conditions characterized by short-term duration or heterogenous recovery patterns [7, 26].

The fifth design choice relates to the surveying techniques. Disability weight data can be collected by focus panel-group discussions or panel meetings, telephone or face-to-face interviews, or web-based or mail/postal surveys using, for example, questionnaire as an instrument.

### Search strategy and eligibility criteria

Following the Preferred Reporting Items for Systematic Reviews and Meta-Analyses (PRISMA) 2020 guidelines [31], in May 2021 we systematically searched electronic databases and search engines namely PubMed (Medline Ovid), Embase, Web of Science, Cochrane, PsycINFO. We also searched for eligible grey literature via other sources (i.e., Google Scholar). Search strings can be found in the Additional file. We registered this systematic literature review protocol on PROSPERO database under ID CRD42021259156.

The inclusion criteria were disability weights measurement studies that derived disability weights for single or multiple health outcomes, published in peer-reviewed journals or grey literature between January 1990 and May 2021. We considered studies assessing disability weights for burden of disease measurements, expressed in DALY estimates. This review included studies that assessed disability weights for multiple health states, since disability weights for one single state of health cannot capture population’s preferences for health states. There were no geographical and language restrictions. We used a translation software for papers in languages we could not read. We excluded studies deriving quality-adjusted life year weights and those deriving disability weights for comparative risk factor assessments (e.g., noise-induced sleep disturbance) as they were beyond the scope of this review.

### Screening and data extraction

After removing duplicates, we selected relevant disability weights measurement studies following three steps. First, we excluded studies on the basis of the title; second, we screened the abstracts of the studies selected in the first step; and third, we read the entire full-texts selected in the second step. During each step, we evaluated the titles, abstracts, or full-texts respectively, using the eligibility criteria described above.

One researcher (PC) performed the screening of data using the EndNote X9 software. PC also handsearched the reference lists of systematic reviews and studies or reports included in this review, in order to detect additional eligible disability weights measurement studies. PC then listed the articles obtained from the databases, search engines, other sources, and reference checks in an Excel spreadsheet, comparing accordingly for eligibility. Two researchers (PC and JH) critically appraised eligible disability weights measurement studies, using the data extraction grid developed for the systematic review by Haagsma et al. [9]. We extracted data relating to the following items: study characteristics and geographical location(s), cause(s) of ill-health outcomes, design choices (i.e. health state description, panel of judges, valuation methods for health states, time presentation, and surveying techniques). PC and JH discussed any disagreements arising from eligibility criteria or data extraction items.

### Data synthesis

Those disability weights measurement studies that we considered for review have been quantitively classified:

- as single-country or multi-country studies based on the geographical location(s) covered;
- as single-cause or multi-cause studies based on the cause(s) of ill-health outcomes for which the disability weights were derived;
- by the methodological design choices that have been used to assess disability weights.

Finally, we plotted the key methodological design choices identified in these studies over period.

### Methodological quality

One researcher (PC) performed the methodological quality of each disability weights measurement study, using a modified version of the Checklist for Reporting Valuation Studies (CREATE) instrument [32]. The quality assessment form can be found in the Additional file. The CREATE checklist aims to promote good reporting practices of methodological design choices in valuation studies. This checklist consists of 21 items grouped in seven domains. For this systematic review, items 1–15 were applicable to all the included studies. However, for the purpose of this review, we modified the items 1, 2, 3, and 15; we also excluded items 16–21, as scoring algorithm and modelling specifications are outside the scope of this review.

## Results

### Literature search

**Fig. 1:**
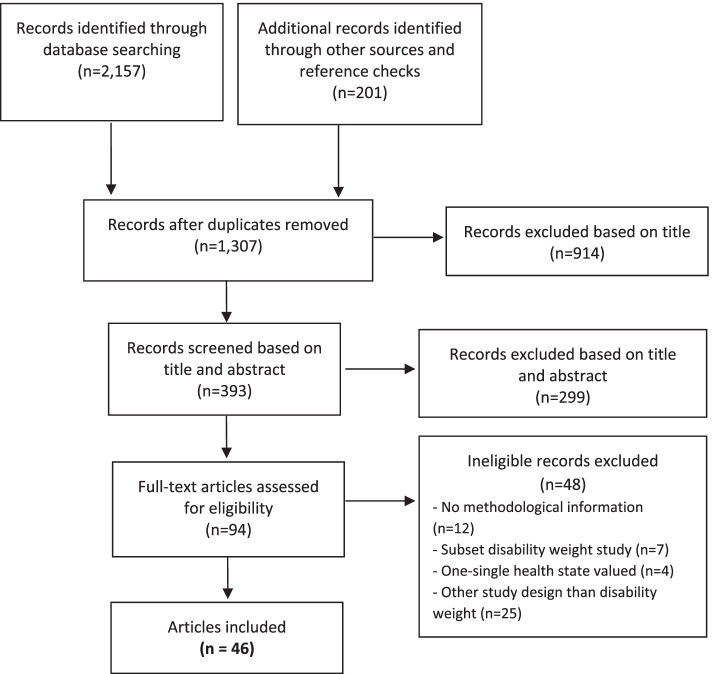
Flow diagram of existing disability weights measurement studies

Figure 1 shows the flow diagram of the search for existing disability weights measurement studies and the main reasons for exclusion. Searches through the electronic databases, search engines, handsearching and the grey literature provided a total of 1307 records. The full-texts of 94 articles were systematically read, and led to the final review of 46 unique disability weights measurement studies.

### Study characteristics

**Fig. 2:**
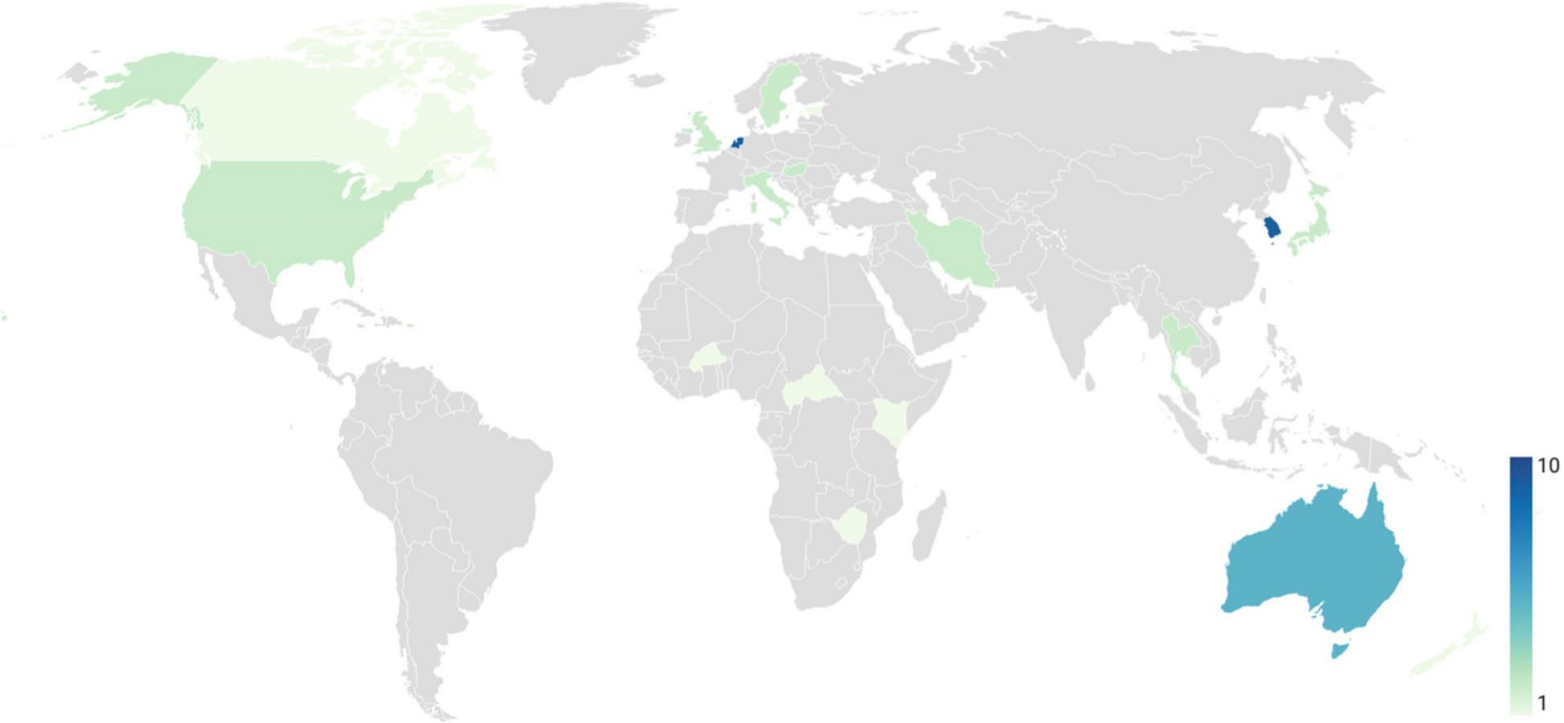
Number of disability weights measurement studies per country A map illustrating the number of studies that estimated disability weights for multiple heath states of disease. Countries in grey indicate that no studies met our eligibility criteria or they have not yet estimated disability weights.

Of the 46 studies included in our systematic literature review, most (*n* = 35; 76%) estimated disability weights at a single-country level, while the remaining 24% (*n* = 11) estimated multi-country disability weights. The single-country disability weights studies were performed across 12 countries. The number of published single-country disability weight studies varied by country, with the lowest number in Estonia (*n* = 1) and Zimbabwe (*n =* 1), and the highest number in South Korea (*n* = 10) and the Netherlands (*n* = 7), (Fig. 2).

**Fig. 3:**
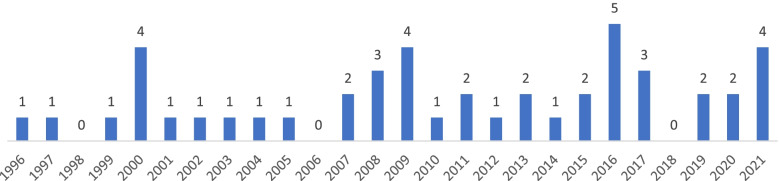
Number of disability weights measurement studies published between 1996 and 2021

As can be seen in Fig. 3, almost every year within the early-1996 to mid-2021 period, one or more than one disability weights measurement studies were published. The earliest study was published in 1996, but none in 1998, 2006, and 2018. The highest number of disability weights measurement studies was seen in 2016 (*n* = 5).

More than half of the identified disability weight measurement studies (*n* = 27; 59%) assessed disability weights for a variety of cause of ill-health outcomes. The remaining nineteen studies (*n* = 19; 41%) concerned disability weights for specific causes or sequelae of diseases (i.e. injuries [33–36], poisonings [37], urological disease [38], periodontal disease [39], oral disease [40], infectious diseases [41], alcohol use disorders [42], mental disorders [43], stroke [44], cardiovascular disease (CVD [45];), multiple sclerosis [46], neoplasms [47, 48], leprosy [17], paediatric congenital anomalies [18], or osteoporosis [49]).

### Methodological quality

Table 1 reports detailed information of the characteristics, methodological and experimental design choices, and methodological quality for each of the 46 disability weights measurement studies. The quality of the included disability weight papers according to the CREATE criteria [32] was very good, with a mean score of 93%. Overall, the major item that did not comply with the CREATE checklist was about stating response rate (66%). All disability weights measurement studies reported on the health state descriptions and valuation techniques, panel of judges, time presentation, study sample, and transformation-modeling analyses.

**Table 1:** Study characteristics, methodological design choices, surveying techniques, and quality of the included disability weights measurement studies

Author	Year	Region or country included	Single- or Multi-cause study?	Panel of judges	Number of the panel members	Number of health states valued	Description of health states	Time Presentation	Valuation methods for health states	Surveying technique/tool	Number of tasks per panel member	CREATE score (%)
Asadi et al. [37]	2016	Iran	Single (Poisonings)	ME	10	50	DS	Period Profile	Interpolation	Questionnaire	NA	93
Bae et al. [49]	2019	Korea	Single (Osteoporosis; osteoporotic fractures)	ME	33	6	NA	Period Profile	PTO	Face-to-face interview	6	87
Bakhshandeh et al. [45]	2009	Iran	Single (Cardiovascular diseases)	HP, PT, PX, PP	80	25	DS	NA	VAS (ranking)	Face-to-face interview	25	80
Baltussen et al. [50]	2002	Burkina Faso	Multiple	HP, PP	56	9	DS (scenario analyses)	Period Profile	Adapted VAS/ Interpolation	Group discussion & Questionnaire	9	93
Basiri et al. [38]	2008	Iran	Single (Urological disease)	ME	10	76	NA	Period Profile	Interpolation	Questionnaire	10	87
Brennan & Spencer [40]	2004	Australia	Single (Oral disease)	Model	NA	18	EQ-5D	Period Profile	EQ-5D: NA	NA	NA	100
Brennan et al. [39]	2007	Australia	Single (Peridontal disease)	Model	NA	6	EQ-5D	Period Profile	EQ-5D: NA	NA	NA	100
Cho et al. [46]	2014	Korea	Single (Multiple sclerosis)	ME	15	5	DS	Period Profile	PTO	Modified Delphi method	NA	87
Choi et al. [47]	2013	Korea	Single (Cancer)	ME	32	24	NA	Period Profile	VAS & PTO	NA	24	87
Gabbe et al. [33]	2016	Netherlands, United States, New Zealand, Australia, United Kingdom	Single (Injury)	Model	NA	40	EQ-5D	Period Profile	EQ-5D: NA	NA	NA	100
Haagsma et al. [34]	2008	Netherlands	Single (Injury)	PP	143	44	DS & EQ-5D	Annual profile	VAS & TTO	Panel meeting & Questionnaire	32	100
Haagsma et al. [51]	2008	Netherlands	Multiple	PP	107	39	DS & EQ-5D	Annual profile	VAS & PTO	Panel meeting & Questionnaire	27	100
Haagsma et al. [35]	2009	Netherlands	Single (Injury)	Model	NA	7	EQ-5D	Period Profile	EQ-5D: NA	NA	NA	100
Haagsma et al. [14]	2015	Hungary, Italy, Netherlands, Sweden	Multiple	PP	30,660	255	DS	Period Profile	PC & PHE	Web-based survey	18	100
Hong & Saver [44]	2009	United States, Southeast Asia	Single (Stroke)	ME	9	5	NA	NA	PTO	Panel meeting & Questionnaire	5	80
Havelaar et al. [41]	2000	Netherlands	Single Infectious disease	ME	35	NA	NA	Period Profile	Interpolation	Panel meeting	NA	93
Jelsma et al. [52]	2000	Zimbabwe	Multiple	ME, PP	68	22	NA	Period Profile	VAS & PR	Focus group discussion	NA	80
Kim et al. [53]	2020	Korea	Multiple	ME	806	313	DS	Period Profile	PR	Web-based survey	20	93
Kruijshaar et al. [54]	2005	Netherlands	Multiple	ME	49	18	DS & EQ-5D	Period Profile	Interpolation	Questionnaire	18	100
Kwong et al. [55]	2010	Canada	Multiple	Model	NA	NA	CLAMES	Period Profile	CLAMES: NA	NA	NA	100
Lai et al. [56]	2009	Estonia	Multiple	ME	25	283	DS	Period Profile	VAS & PTO	Panel meeting	NA	93
Lyons et al. [36]	2011	United Kingdom	Single (Injury)	Model	NA	13	EQ-5D	Period Profile	EQ-5D: NA	NA	NA	93
Mathers et al. [57]	2000	Australia	Multiple	Model	NA	NA	EQ-6D & DDW	Period Profile	EQ-6D: NA	NA	NA	100
Murray et al. [8]	1996	Global	Multiple	ME	10	483	DS	Period Profile	VAS & PTO	NA	NA	87
Nanjan Chandran et al. [17]	2021	Global	Single (Lepropsy)	PP	667	3	SF-36 HRQoL data	NA	SF-36 HRQoL data: NA (meta-analysis)	NA	NA	100
Neethling et al. [16]	2016	South Africa	Multiple	PP	677	51	DS (no label)	Period Profile	PC & TTO	Household face-to-face interview	15	100
Nontarak et al. [58]	2020	Thailand	Multiple	PT	450	9	DS & EQ-5D	Period Profile	VAS & EQ-5D & TTO	Face-to-face interview	NA	87
Nontarak et al. [42]	2021	Thailand	Single (Alcohol use disorders)	PT, PP	300	3	DS & EQ-5D	Period Profile	VAS & EQ-5D & TTO	Face-to-face interview	NA	93
Nomura et al. [13]	2021	Japan	Multiple	PP	37,318	231	DS (no label)	Period Profile	PC & PHE	Web-based survey	18	100
Ock et al. [59]	2016	Korea	Multiple	HP	496	228	DS	Period Profile	PC & PTO	Web-based survey	60	93
Ock et al. [15]	2016	Korea	Multiple	PP	5916	256	DS	Period Profile	PC & VAS & SG & PHE	Household survey & Web-based survey	22	93
Ock et al. [60]	2019	Korea	Multiple	HP	605	289	DS (no label)	Period Profile	PR	Web-based survey	20	93
Park & Jung [61]	2017	Korea	Multiple	Model	NA	243	EQ-5D	NA	EQ-5D: NA	NA		100
Piao et al. [62]	2021	Japan	Multiple	HP	286	17	DS	Period Profile	PC & PHE	Questionnaire	NA	87
Poenaru et al. [18]	2017	Kenya, Canada	Single (Paediatric conditions)	HP	154	15	DS & EQ-5D	NA	PR & VAS & PC & TTO	Focus groups	NA	87
Rehm & Frick [63]	2013	United States	Multiple	ME	68	12	DS + CLAMES	NA	PC & PR & PTO	NA	NA	87
Salomon et al. [12]	2012	Global	Multiple	PP	30,230	220	DS (no label)	Period Profile	PC & PHE	Household survey; Web-based survey	18	100
Salomon et al. [20]	2015	Global	Multiple	PP	30,230 & 30,660	235	DS (no label)	Period Profile	PC	Web-based survey (GBD 2010; European surveys; Household surveys; USA surveys)	18	100
Sanderson et al. [43]	2001	Australia	Sinlge (Mental disorders)	ME	20	19	DS	NA	PTO & Rating scale & PR	Panel meeting	19	87
Schwarzinger et al. [64]	2003	Europe	Multiple	ME, nHP	232	15	DS & EQ-5D	Period Profile	VAS & PTO & TTO	Panel meeting	15	93
Steckling et al. [19]	2017	Global	Multiple	ME	18	105	DS & EQ-5D	Period Profile	VAS & PC/ Interpolation	Web-based survey	NA	87
Stouthard et al. [65]	1997	Netherlands	Multiple	ME	38	175	DS & EQ-5D	Period Profile & Annual Profile	VAS & PTO	Panel meeting & Questionnaire	47	93
Ustün et al. [66]	1999	Global	Multiple	HP, PT, PX, PM	241	17	DS	Period Profile	PR	Interview	17	93
Van Spijker et al. [67]	2011	Netherlands	Multiple	ME	16	12	DS & EQ-5D	Period Profile	VAS	Questionnaire	12	100
Yoon et al. [48]	2000	Korea	Single (Cancer)	ME	19	12	NA	NA	PTO & Interpolation	Delphi method	NA	80
Yoon et al. [68]	2007	Korea	Multiple	ME	30	123	NA	Period Profile	PTO & Interpolation	Panel meeting	53	93

*NA* Not Available, *DDW* Dutch Disability Weight

Panel of judges

*ME* Medical experts, *HP* Health professionals, *nHP* Non-health professionals, *PT* Patients or people with disabilities, *PX* Patient proxies, *PM* Policy-makers, *PP* Population

Description of health states

*DS* Disease-specific health state description, *EQ-5D* European Quality of Life instrument that consists of five dimensions/attributes (mobility, self-care, usual activities, pain/discomfort, and anxiety/depression), *EQ-6D* EQ-5D appended with a cognitive dimension/attribute, *CLAMES* Classification and Measurement System of Functional Health, *SF-36 HRQoL* Short form health-related quality of life survey

Valuation methods for health states

*VAS* Visual analog scale, *TTO* Time trade-off *PTO* Person trade-off, *PC* Paired comparison, *PHE* Population health equivalence, *PR* Preference ranking, *SG* Standard gamble

CREATE

CREATE: Checklist for Reporting Valuation Studies instrument

### Methodological design choices

#### Description of health states

Seven disability weights measurement studies (*n* = 7; 15%) used validated multi-attribute utility instruments [33, 35, 36, 39, 40, 55, 61]; such health-related instruments use preferences to develop norms for health states of disease. Six of these studies (*n* = 6) used the EQ-5D model [33, 35, 36, 39, 40, 61], while one study (*n* = 1) assessed disability weights for health conditions using the CLAMES methodology [55]. Moreover, a systematic review and meta-analysis of individual patient data obtained new estimates of leprosy disability weights based on SF-36 health-related quality of life data [17]. Thirty disability weights measurement studies (*n* = 30; 65%) described the health states using the disease-specific system [8, 12–16, 18–20, 34, 37, 42, 43, 45, 46, 50, 51, 53, 54, 56–60, 62–67]. In these studies, the disease-specific health states were presented in terms of brief lay descriptions (or without label), or disability weight scenario analyses or a combination of a disease-specific description of health effects and generic instrument information. Eight studies did not report on the health state description system for the diseases that were valued [38, 41, 44, 47–49, 52, 68].

**Fig. 4:**
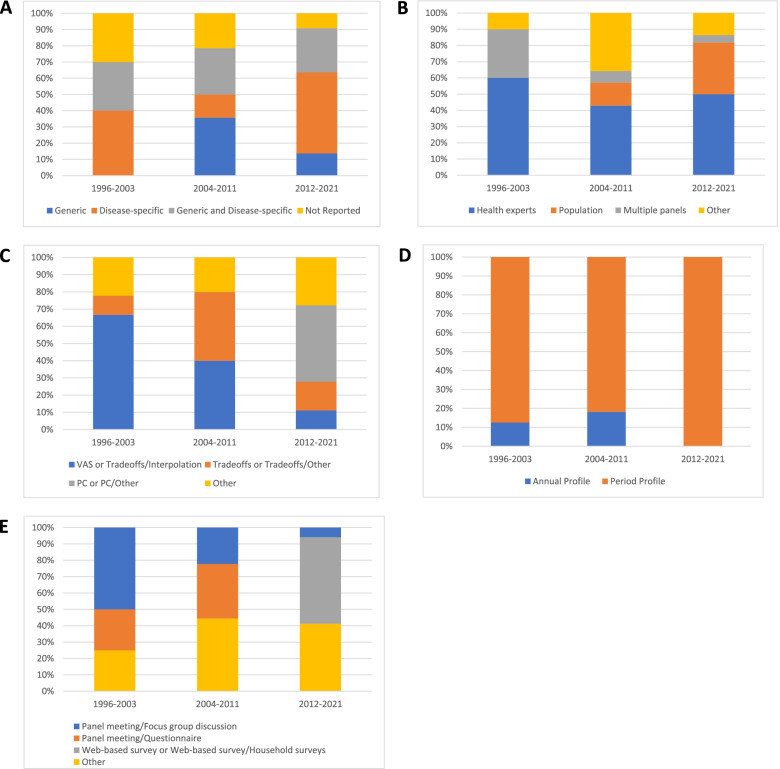
Evolution of methodological design choices in disability weights measurement studies: (**A**) Description of health states, (**B**) Panel of judges, (**C**) Valuation methods for health states, (**D**) Time Presentation, and (**E**) Surveying technique

Around 30% of the disability weights measurement studies that were published during each period (i.e. 1996–2003, 2004–2011, and 2012–2021) used a combination of generic and disease-specific health descriptions to assess disability weights (Fig. 4 A). However, over the 2012–2021 period, half (50%) of the identified studies used disease-specific methods to depict health states of disease, a similar percentage to that of the 1996–2003 period (Fig. 4 A).

The majority of the studies (53%) did not report on the process of evaluating the validity of health state descriptions. Some studies, however, reported that lay descriptions of health states were circulated to disease experts or health professionals for face validation purposes [12–14, 18, 20, 46, 50, 64, 65].

Notably, the number of health states valued in the included disability weights measurement studies varied from three [17] to 483 [8].

#### Panel of judges

Among the studies that did not estimate disability weights using multi-attribute utility instruments, 59% (*n* = 22) included panels of medical or clinical experts or health professionals [8, 18, 19, 37, 38, 41, 43, 44, 46–49, 53, 54, 56, 59, 60, 62, 63, 65, 67, 68]. Nine studies obtained health state preferences from a general population panel [12–17, 20, 34, 51], whereas six studies included more than one panel of judges [42, 45, 50, 52, 58, 66]. Specifically, Baltussen et al. [50] obtained disability weights based on general population and health professionals’ preferences and found that health professionals rated seven out of nine states of health as slightly to moderately less severe compared to lay people from the general population. A study conducted by Jelsma et al. [52] included medical experts’ and population preferences for multiple health states and showed strong differences among lay people and medical experts. Bakhshandeh et al. [45] showed differences between CVD disability weights obtained from patients, patients’ families, health professionals, and health professionals. Schwarzinger et al. [64] reported on the agreement level of disability weights among five Western European countries based on health professionals’ and non-health professionals’ preferences and showed a lower level of agreement in the cases of PTO disability weights and higher level of agreement in the cases of VAS and TTO disability weights. Nontarak et al. [42] found differences in disability weight estimates between patient and non-patient population preferences. Ustün et al. [66] showed significant differences in ranking of health conditions across 14 countries. Notably, Nontarak et al. [58] derived patients’ self-reported disability weights.

Additionally, the percentage of disability weight studies obtaining health preferences from a population-based panel increased from 14% (2004–2011) to 32% (2012–2021). In general, the percentage of studies that derived disability weights from a panel of health experts slightly decreased (Fig. 4 B).

The lowest number of judges identified in disability weight studies was nine [44]. The largest number of judges was seen in the Salomon et al. [20] study, a combined sample size consisting of 30,230 respondents from the GBD 2010 household surveys and 30,660 from the European disability weights measurement study.

#### Valuation methods for health states

Of the disability weight studies that did not use a multi-attribute utility instrument, 32% (*n* = 12) obtained health state preferences using trade-off or VAS methods (first step) and interpolation tasks (second step) [8, 19, 37, 38, 41, 48, 50, 54, 56, 64, 65, 68]. However, some studies combined a PC approach with other valuation techniques for health states [12–16, 20, 59, 62], whereas other studies used only trade-off [34, 44, 46, 49, 51] or rank [45, 52, 53, 60, 66] or VAS approach [67] to value the health states of disease.

The percentage of studies that followed a two-step approach to value health state preferences was higher during the 1996–2003 period, rather than the 2004–2011 and 2012–2021 periods (Fig. 4 C). After the 2004–2011 period, more and more disability weight studies used PC techniques to assess disability weights rather than trade-off tasks.

#### Time presentation

All disability weights measurement studies used the period profile approach. Three Dutch disability weights (DDW) studies [34, 51, 65] used the annual profile approach.

None of the disability weight studies published in the past 10 years used the annual profile approach (Fig. 4 D).

#### Surveying techniques

We identified several surveying techniques in disability weights measurement studies (Table 1). Most studies performed meetings or focus-group discussions with the panel of judges [18, 41, 43, 52, 56, 64, 68] or a combination of group discussions and individual questionnaires [34, 44, 50, 51, 65]. Six studies used web-based surveys to collect the data [13, 14, 19, 53, 59, 60]. Other studies performed interviews [42, 45, 58, 66]. Two studies obtained disability weights data using the Delphi method [46, 48]. Mixed surveying techniques were used in the GBD 2010 disability weights study (face-to-face or telephone survey and a web-based survey [12]) and in the South Korean disability weights study (household survey involving computer-assisted face-to-face interviews and a web-based survey [15]).

Between 1996 and 2013, half (50%) of the identified studies collected disability weight data by performing panel meetings of focus-group discussions (Fig. 4 E). Over the years, however, these surveying techniques have been eliminated, with web-based surveys or both web-based and household surveys (53%) appearing during the 2012–2021 period.

## Discussion

### Summary of findings and interpretation of results

This systematic literature review has provided insights into the methodological design choices that have been made to describe and value health states in disability weights measurement studies. We aimed to provide an update on studies estimating disability weights between the early-1996 and mid-2021 period. We gathered methodological approaches and surveying techniques from 46 unique disability weights measurement studies and we studied how these key design choices evolved over time.

Health state descriptions are an important matter in disability weights measurement studies. We found that half of the included studies published between 2012 and 2021 had used disease-specific descriptions in line with those of the GBD study. In general, from early-1996 to mid-2021, we observed an increased number of national disability weights studies using the GBD lay descriptions to depict each cause of the health states. This corresponds to validity, consistency, and therefore similar patterns of disability weights between national and GBD disability weights measurement studies. Additionally, a variety of disability weights studies (2012–2021) had used a combination of disease-specific and generic-preference instruments to describe and value states of health, compared to those published during the 1996–2003 and 2004–2011 periods. Although there are differences between those design choices, both can be applied to quantify the severity of a particular health state. However, describing health using generic instruments may result in information loss as the disease-specific symptoms are not described. Thus, generic health state descriptions are recommended to be used in combination with disease-specific descriptions to strengthen the standardization of the health state description system.

A noteworthy observation of this review is that, after 2010, the percentage of disability weights measurement studies deriving preferences from general population panels had more than doubled. Disability weights may be affected by the choice of the panel composition [69, 70]. Individual preferences obtained from patients differ from those of the population. It has also been shown that disability weight values differ between medical or health experts and the general population [45, 50, 52, 64, 66]. However, population-based panels can yield valid disability weight estimates as opposed to preferences obtained from patients or health professionals [71]. Driven by the fact that burden of disease studies is an important tool for decision-making processes and setting health priorities for populations, it is important to incorporate general populations’ perceptions [12, 71]. However, when the panel of judges consists of members of the general public, this may also mean that valid health state valuation data are more difficult to obtain. Since the general population often has no knowledge of or experience with the presented disease or health state itself, it is paramount to develop health descriptions that are valid and understandable to lay persons. Our study showed that the process of evaluating the validity of health state descriptions in disability weights measurement studies was often not reported.

Moreover, we identified a large variation in the size of the panel of judges. Based on the performed methodological quality assessment, we found a gap in the reporting of the calculation of the size of the panel. The size of the panel depends on the number of health states included for valuation and on the minimum number of observations per health state that is set by the researchers. However, the minimum number of observations per health state was often not reported. This might call for improvements in the reporting of future disability weights measurement studies.

Apart from the minimum number of observations per health state, the size of the panel also depends on the number of valuation tasks that each individual panel member performs. Our findings showed that the number of tasks per individual range from five [44] to 60 [59]. However, is highly important to take into account the aforementioned choice, as the vast majority of panel members will not be familiar with the health state valuation tasks, particularly in case of panels that consist of members of the general public. If the number of tasks per person is too small, the panel members will not be able to familiarize themselves with the task and gain an understanding of the tasks. On the other hand, if the number of tasks per person is too high, response fatigue may increase. Both may impact the quality of the health state valuations considerably.

Another finding of this review is that the majority of disability weights measurement studies used one or more than one valuation method to elicit preferences. However, most multi-country but also some single-country studies, conducted after 2012, estimated disability weights using the PC in combination with the PHE and/or the VAS techniques. However, two disability weights studies that used PHE to assess preferences from a general population sample showed that the quality of the PHE data was low and could not be used for the calculations of the disability weights [13, 14]. This indicates that the use of the PHE is most likely too complex to be used in a general population setting and more simplified valuation methods should be used in future disability weight studies in a similar setting and with similar surveying techniques. Other methodological applications have been developed, such as the DELPHI processes applied in two Korean disability weights studies [46, 48]; DELPHI technique allows for structured panel-group communication in order to deal with complex issues where knowledge is uncertain or incomplete [72]. An essential step in disability weights measurement studies is to transform health state valuation data into a disability weight that is anchored between 0 and 1. For cardinal methods, such as the VAS and TTO, this step is easier compared to ordinal methods, such as the PC. A review of mathematical methods that were used to transform health state valuation data into disability weights is out of the scope of our study. However, it is highly important that disability weights studies clearly describe the procedure that is followed to calculate disability weights from health state valuation data to improve reproducibility and comparability of disability weights measurement studies. Development of more detailed reporting guidelines for the transformation of health state valuation data into disability weights or health state utilities may facilitate reproducibility and comparability.

Additionally, the results of our systematic review showed that very few studies assessed annual profile disability weights and that over years the period health profile approach has been adopted more often. Several reasons can be discussed regarding the limited application of the annual health profile approach. First, it might not be feasible for panellists to imagine living a short-term condition over a period of 1 year as the annual profile approach assumes constant health over one full year. Second, it has been argued that the use of annual profile disability weights in burden of disease assessments would give undue weight to conditions with a mild and rapid course [73].

Moreover, most disability weights measurement studies (1996–2003) performed panel meetings or focus group discussions as surveying techniques, whereas from 2012 onwards household surveys and/or web-based surveys have frequently been used. The latter technique, may elicit selection bias, since internet users are over-represented among the study-participants. Another reason for this bias may be that individuals with a higher level of education use the internet more frequently than individuals with a lower level of education [74]. To overcome this bias, we recommend the selection of panels with certain characteristics (i.e. age, sex, socio-demographic information, or cultural background). Notably, a study conducted by Jelsma et al. suggests that cultural differences on valuations may have a strong effect among lay people compared to health experts [52].

Coverage of causes of disease and injury in different health states differs markedly among the multi-cause disability weights measurement studies. The GBD 1996 [8], the Estonian [56] and the updated Korean [60] set of disability weights cover a variety of health conditions compared to the DDW study [65]; however, the DDW study, on the other hand, provides a more detailed differentiation between disease stages, severities, treatment, and prognosis [65]. This allows more consistent modelling approaches when quantifying the burden of disease. Among the single-cause disability weights studies, we observed that more specific stages of disease are included. These studies were conducted either to develop disability weights that are not yet available from the GBD study effort (e.g., wrist osteoporotic fractures [49], chronic metallic mercury vapor intoxication [19] etc) or to estimate disability weights that were not available from the GBD study and have been applied in its latest iterations (e.g., harmful alcohol disorders [42], concussion [34], irritable bowel syndrome [51] etc).

Assessing the validity of disability weights is not an easy task as there is no gold standard for disability weights [9]. However, various methodological approaches have been suggested to evaluate the validity of disability weights. First, comparing the ranking of disability weights between similar studies and/or detecting if the disability weights of diseases or injuries increase according to their severity level (i.e., mild, moderate, severe) [9, 53, 60]. The latter approach tallies with the assessment of face validity and is therefore recommended to be used in future disability weights measurement studies. Second, Maertens de Noordhout et al. [75] suggested to compare EQ-5D’s DWs with utility weights; hence, utilization of EQ-5D health states in order to evaluate the validity of the disability weights has been previously applied [15].

### Strengths and limitations of the study

An important limitation associated with this systematic literature review is that only one source was considered for grey literature searches. There is also a risk for publication bias because we did not search other languages than English. Moreover, it is possible that other disability weights measurement studies have been conducted but not published. Despite these limitations, we emphasize that this systematic literature review provides an extensive overview for understanding the methodological design choices and surveying techniques that were used in disability weights measurement studies. This review showed that from 1996 to 2021, the national disability weight applications have led to substantial changes in design choices and surveying techniques, allowing for comparability of the disability weight values. Finally, we sought to provide recommendations that may help to design and develop future disability weights measurement studies but also to evaluate the validity of disability weights.

## Conclusions

Our systematic literature review reveals that a methodological uniformity between national and GBD disability weights measurement studies increased, especially from 2010 onwards. This uniformity relies on the health state descriptions, the choice of the panel composition, the time presentation, and the surveying techniques. However, in terms of valuation techniques that have been used to describe and value disability weights, there is a wide variation in national disability weights studies that persisted over time.

## Supplementary Information

**Additional file 1.**

## Data Availability

Not applicable.

## Abbreviations

CLAMES: Classification and Measurement System of Functional Health
CREATE: Checklist for Reporting Valuation Studies
CVD: Cardiovascular Disease
DALY: Disability-Adjusted Life Year
DDW: Dutch Disability Weights
DS: Disease-specific
EQ-5D: European Quality of Life instrument – 5 Dimensions (mobility, self-care, usual activities, pain/discomfort, and anxiety/depression)
EQ-6D: EQ-5D appended with a cognitive dimension
GBD: Global Burden of Disease
HRQoL: Health-Related Quality of Life
HP: Health Professionals
ME: Medical Expert
NA: Not Available
nHP: Non-Health Professionals
PC: Paired Comparison
PHE: Population Health Equivalence
PM: Policymakers
PP: Population
PR: Preference Ranking
PT: Patients or people with disabilities
PX: Patient proxies
PTO: Person Trade-Off
SF-36: Short form health-related quality of life survey
SG: Standard Gamble
TTO: Time Trade-Off
VAS: Visual Analogue Scale
YLD: Years Lived with Disability
YLL: Years of Life Lost
