# Supplementary material for: A systematic literature review of disability weights measurement studies: evolution of methodological choices
Source: Arch Public Health. 2022 Mar 24;80:91. doi: 10.1186/s13690-022-00860-z (PMC8944058; doi:10.1186/s13690-022-00860-z)
Supplement: Supplementary file 1 — Additional file 1. [file 13690_2022_860_MOESM1_ESM.docx]

**Appendix file**

*Supplement to*: Charalampous P, Polinder S, Wothge J, von der Lippe E, Haagsma JA. A systematic literature review of disability weights measurement studies: evolution of methodological choices

1. Search strategy .…………………………………………………………………………………………………………………….…… 2
2. Methodological quality assessment ….……………………………………………………………………………………..… 3
3. Methodological choices to derive disability weights: a summary ……………………………………………..… 4
4. **Search strategy**

Embase.com

('disability weight'/de OR (((disabilit* OR severit*) NEXT/1 (weight OR weights))):ab,ti,kw) AND [1990-2021]/py

Medline Ovid

1. ((((disabilit* OR severit*) ADJ (weight OR weights))).ab,ti,kf.)

2. Limit 1 to yr=1990-2021

PsycINFO Ovid

1. ((((disabilit* OR severit*) ADJ (weight OR weights))).ab,ti.)

2. Limit 1 to yr=1990-2021

Web of Science

TI=(((disabilit* OR severit*) NEAR/1 (weight OR weights))) OR AB=(((disabilit* OR severit*) NEAR/1 (weight OR weights))) AND PY=1990-2021

Cochrane Central

((((disabilit* OR severit*) NEXT/1 (weight OR weights))):ab,ti,kw)

Google Scholar

"disability|severity weight|weights"

1. **Methodological quality assessment**

- In this systematic review an adjusted version of the Checklist for Reporting Valuation Studies of Multi-Attribute Utility-Based Instruments (CREATE)* was used.
- Red-coloured text indicates the adjustments made. Please note that the authors decided to not consider items 16-21.

| **Item no.** | **Section/item** | **Yes** | **No** |
| --- | --- | --- | --- |
| Descriptive system | | | |
| 1 | The attributes of the instrument are described  *The number of health state descriptions is described* | □ | □ |
| 2 | The number of levels in each attribute of the instrument is described  *The health state descriptions are described* | □ | □ |
| Health states valued | | | |
| 3 | The approach to selecting health states to be valued directly is explained  *The approach to selecting health states to be valued per valuation technique is explained* | □ | □ |
| 4 | The number of health states valued per respondent is stated | □ | □ |
| 5 | Method(s) of assigning the health states to respondents are stated | □ | □ |
| Sampling | | | |
| 6 | Sample size/power calculations are stated and rationalized | □ | □ |
| 7 | Target population is described | □ | □ |
| 8 | Sampling method is stated and rationalized | □ | □ |
| 9 | Recruitment strategies are described | □ | □ |
| 10 | Response rate is reported | □ | □ |
| Preference data collection | | | |
| 11 | Mode of data collection is stated | □ | □ |
| 12 | Preference elicitation technique(s) are described | □ | □ |
| Study sample | | | |
| 13 | Reasons for excluding any respondents or observations are provided | □ | □ |
| 14 | Characteristics of respondents included in the analysis are described | □ | □ |
| Modeling | | | |
| 15 | The dependent variable for each model is stated  *The statistical methods to transform the preference data into disability weights is described* | □ | □ |
| ~~16~~ | Independent variables for each model are explained | □ | □ |
| ~~17~~ | Model specifications are provided | □ | □ |
| ~~18~~ | Model estimators are described | □ | □ |
| ~~19~~ | Goodness-of-fit statistics for each model are reported | □ | □ |
| Scoring algorithm | | | |
| ~~20~~ | Criteria for selecting the preferred model are stated | □ | □ |
| ~~21~~ | The scoring algorithm is presented | □ | □ |

* Xie F, Pickard AS, Krabbe PF, Revicki D, Viney R, Devlin N, Feeny D: **A Checklist for Reporting Valuation Studies of Multi-Attribute Utility-Based Instruments (CREATE).** *Pharmacoeconomics* 2015, **33:**867-877

1. **Methodological choices to derive disability weights: a summary**

- We have sought to summarize the strengths and weaknesses of the methodological design choices that have been used to derive disability weights.
- The following methodological aspects might be useful in future disability weights measurement studies.

| **Methodological design choice** | **Summary of findings** |
| --- | --- |
| Health state description | - Generic health state descriptions in combination with disease-specific descriptions strengthen the standardization of the health state description system. - When presenting disease-specific lay descriptions, it is paramount to check whether the health state descriptions are valid and understandable to lay persons. Study materials/handouts can be evaluated by disease experts or health professionals beforehand. This corresponds to face validity, an important aspect of disability weights studies. |
| Panel of judges | - Our study showed that more and more disability weights studies obtained health preferences from a population-based panel. This highlights even more the need to develop valid health descriptions, as the general population lacks knowledge about the disease itself. - Disability weights measurement studies obtaining preferences from medical experts or health professionals were seen to decrease over time. |
| Valuation methods | - Our study showed that more and more studies used paired comparison tasks to value health states. Over the years, the use of more complex valuation techniques, such as trade-offs, was seen to decrease. - It follows that conceptually less difficult valuation techniques (i.e., visual analog scale or paired comparisons instead of trade-offs) might be more understable to population-based panels. |
| Time presentation | - Our study showed an increase in disability weights studies assessing period profile disability weights. - Period profile disability weights assume independence between duration and disability, meaning that the health state remains constant over time. - This assumption is however untenable for disorders that are characterized by a complex time-severity course; thus, the annual profile approach can be applied. |
| Surveying techniques | - In recent years, most of the included disability weights studies developed web-based sample surveys to collect disability weights data. |
